# Supplementary material for: Analysis of Chemical Composition and Biological Activities of Essential Oils from Different Parts of Alpinia uraiensis Hayata
Source: Molecules. 2025 Mar 28;30(7):1515. doi: 10.3390/molecules30071515 (PMC11990677; doi:10.3390/molecules30071515)
Supplement: Supplementary file 1 [file molecules-30-01515-s001.zip › Supplementary Materials_Table S2.pdf]

Table S2. Antifungal activity of essential oils from different parts of *A. uraiensis* against *P. noxius.* (original raw data)

Concentration	Antifungal index (%)				
(μg/mL)	Leaf	Flower	Stem	Rhizome	Root
800	33.3 ± 4.4	56.0 ± 0.8	28.6 ± 4.4	32.6 ± 4.6	-
400	26.4 ± 5.8	41.5 ± 3.0	19.2 ± 6.8	23.9 ± 4.1	-
260	-	-	-	-	43.3 ± 2.5
200	19.7 ± 5.2	29.6 ± 5.1	17.2 ± 6.4	18.1 ± 1.7	-
130	-	-	-	-	33.9 ± 3.8
100	16.1 ± 4.1	26.4 ± 3.6	16.1 ± 4.7	16.1 ± 5.3	-
65	-	-	-	-	41.3 ± 1.8
50	14.3 ± 5.0	16.5 ± 4.3	11.4 ± 1.2	13.9 ± 1.6	-
32.5	-	-	-	-	23.3 ± 4.3
16.25	-	-	-	-	15.7 ± 5.0
